# Assessing reproducibility and utility of clustering of patients with type 2 diabetes and established CV disease (SAVOR -TIMI 53 trial)

**DOI:** 10.1371/journal.pone.0259372

**Published:** 2021-11-19

**Authors:** Yasunori Aoki, Bengt Hamrén, Lindsay E. Clegg, Christina Stahre, Deepak L. Bhatt, Itamar Raz, Benjamin M. Scirica, Jan Oscarsson, Björn Carlsson

**Affiliations:** 1 Clinical Pharmacology and Safety Sciences, AstraZeneca, Gothenburg, Sweden; 2 Clinical Pharmacology and Safety Sciences, AstraZeneca, Gaithersburg, MD, United States of America; 3 Late-Stage Development, Cardiovascular, Renal and Metabolism (CVRM), BioPharmaceuticals R&D, AstraZeneca, Gothenburg, Sweden; 4 Brigham and Women’s Hospital Heart & Vascular Center, Boston, MA, United States of America; 5 Harvard Medical School, Boston, MA, United States of America; 6 Hadassah University Hospital, Jerusalem, Israel; 7 Research and Early Development, Cardiovascular, Renal and Metabolism (CVRM), BioPharmaceuticals R&D, AstraZeneca, Gothenburg, Sweden; Faculdade de Medicina de São José do Rio Preto, BRAZIL

## Abstract

**Objective:**

To assess the reproducibility and clinical utility of clustering-based subtyping of patients with type 2 diabetes (T2D) and established cardiovascular (CV) disease.

**Methods:**

The cardiovascular outcome trial SAVOR-TIMI 53 (n = 16,492) was used. Analyses focused on T2D patients with established CV disease. Unsupervised machine learning technique called “k-means clustering” was used to divide patients into subtypes. K-means clustering including HbA1c, age of diagnosis, BMI, HOMA2-IR and HOMA2-B was used to assign clusters to the following diabetes subtypes: severe insulin deficient diabetes (SIDD); severe insulin-resistant diabetes (SIRD); mild obesity-related diabetes (MOD); mild age-related diabetes (MARD). We refer these subtypes as “clustering-based diabetes subtypes”. A simulation study using randomly generated data was conducted to understand how correlations between the above variables influence the formation of the cluster-based diabetes subtypes. The predictive utility of clustering-based diabetes subtypes for CV events (3-point MACE), renal function reduction (eGFR decrease >30%) and diabetic disease progression (introduction of additional anti-diabetic medication) were compared with conventional risk scores. Hazard ratios (HR) were estimated by Cox-proportional hazard models.

**Results:**

In the SAVOR-TIMI 53 trial based dataset, the percentage of the clustering-based T2D subtypes were; SIDD (18%), SIRD (17%), MOD (29%), MARD (37%). Using the simulated dataset, the diabetes subtypes could be largely reproduced from a log-normal distribution when including known correlations between variables. The predictive utility of clustering-based diabetic subtypes on CV events, renal function reduction, and diabetic disease progression did not show an advantage compared to conventional risk scores.

**Conclusions:**

The consistent reproduction of four clustering-based T2D subtypes can be explained by the correlations between the variables used for clustering. Subtypes of T2D based on clustering had limited advantage compared to conventional risk scores to predict clinical outcome in patients with T2D and established CV disease.

## Introduction

Type 2 diabetes (T2D) is a heterogeneous syndrome with a large variation between individuals in disease progression and risk of complications. It would be desirable to subtype patients according to their underlying pathophysiology and thereby tailor treatment strategies for better disease control and reduced risk of complications. To address this, Ahlqvist et al. [1] proposed to subgroup patients with newly diagnosed adult-onset diabetes using k-means clustering [2] utilizing predefined diabetes biomarkers and patient characteristics selected based on current clinical and mechanistic understanding of diabetes including homoeostatic model assessment 2 (HOMA2) of insulin resistance (HOMA2-IR) and beta-cell function (HOMA2-B), HbA1c, age at diabetes diagnosis, BMI, and presence of glutamic acid decarboxylase autoantibody (GADA). Five clusters were identified of which GADA defined a cluster as severe autoimmune diabetes (SAID). The remaining four clusters that included type 2 diabetes were: severe insulin deficient diabetes (SIDD); severe insulin resistant diabetes (SIRD); mild obesity-related diabetes (MOD); mild age-related diabetes (MARD). This clustering-based diabetes subtyping approach has been shown to be robust and reproducible in different diabetes populations [3–9].

To further investigate the characteristics and utility of clustering of patients with diabetes according to Ahlqvist et al., we applied k-means clustering using the variables specified by Ahlqvist et al., to a patient cohort with T2D and established cardiovascular (CV) disease from the SAVOR-TIMI 53 trial [10].

We hypothesized that the k-means clustering, using pre-defined diabetes variables [1], in the SAVOR population with established CV disease will reproduce similar patient subtypes as described by Ahlqvist et al. [1] and others [3–5, 7, 8]. We further hypothesized that the consistent reproduction of the patient subtypes can be explained by correlations between the variables used for clustering. Assuming the subtype construction relied on already known correlations between the diabetes related variables, we further hypothesized that the predictive strength of diabetes subtypes is similar to conventional risk scores for diabetic disease progression or complications in high risk patients with T2D and established CV disease.

## Research design and methods

### Patients and data used—SAVOR-TIMI 53 trial-based dataset

To investigate the reproducibility of clustering-based diabetes subtypes and their clinical utility in patients with T2D and established CV disease, we used the dataset from the cardiovascular outcome phase-IV trial SAVOR-TIMI 53 [10], including both active and placebo arms (n = 16,492). SAVOR-TIMI 53 investigated the effect of saxagliptin on CV outcome in patients with T2D and high CV risk. In our post-hoc analysis, both arms were included since there was no effect of active treatment on the primary MACE endpoint, or on eGFR or renal outcomes such as doubling of creatinine or initiation of renal replacement therapy [11]. According to the protocol, patients with type 1 diabetes were excluded. In addition, patients with current or previous use of DPP4 inhibitors or GLP-1 analogues, patients with recent (less than 2 months prior to the randomization) acute cardiac or stroke event, patients with severe renal disease (chronic dialysis and/or renal transplant and/or serum creatinine >6.0 mg/dL) were excluded from the trial.

**Fig 1:**
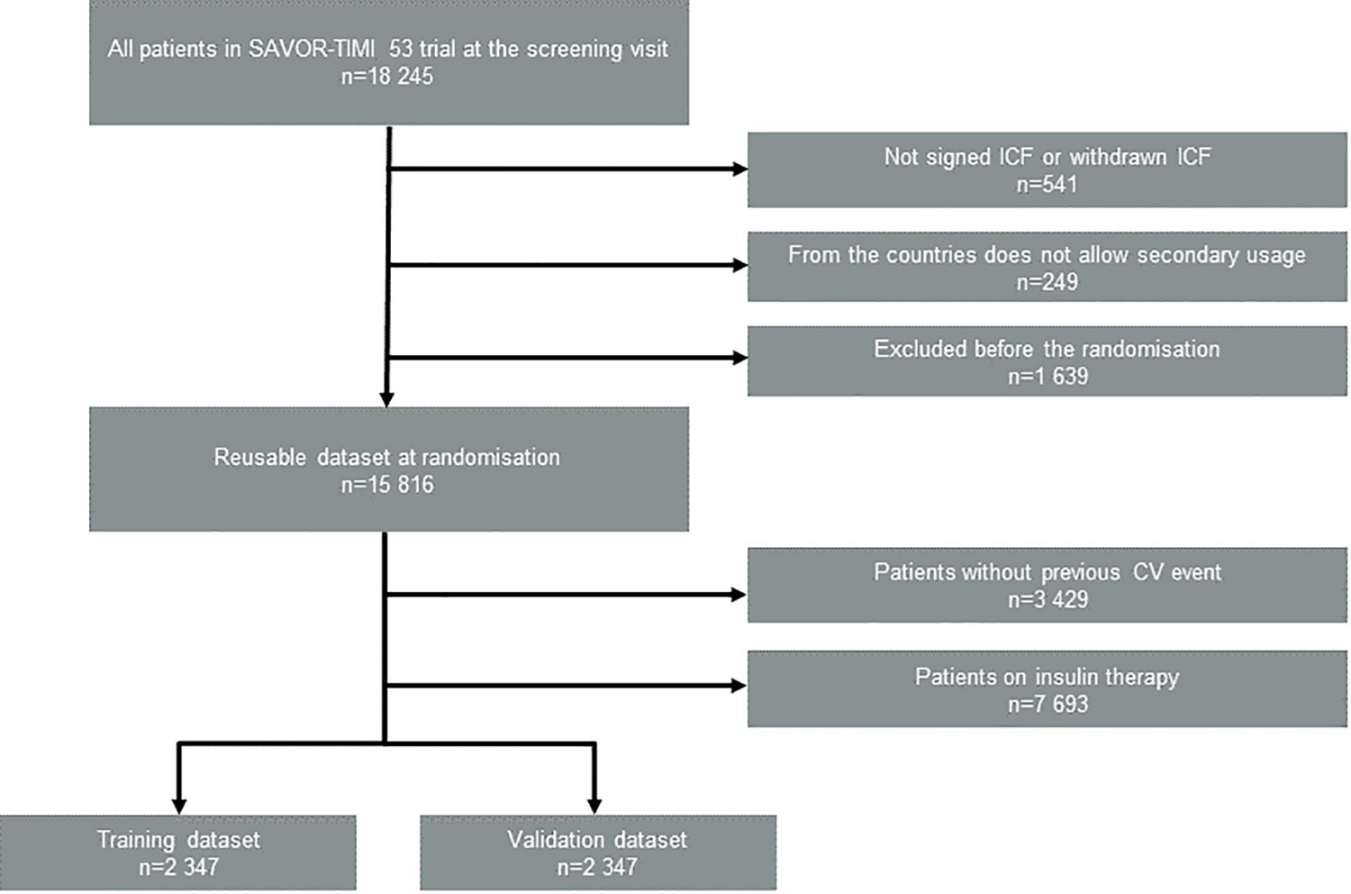
Patient disposition flow diagram for the dataset for our secondary analysis.

In order to focus our attention on the patient subpopulation at risk for recurrent MACE events, we included the patients with established CV disease (ischemic heart disease and/or peripheral vascular disease (e.g., intermittent claudication), and/or ischemic stroke) as defined in the protocol. We excluded patients who were on insulin therapy in order to accurately use HOMA2 values, which are based on fasting serum insulin (f-Insulin) levels. As a result, 4694 patients out of 16,492 in the total population were included in our clustering analysis (Fig 1). This subgroup of patients had a mean follow-up of 2.1 years and mean diabetes duration of 8.7 years. We further divided this patient subpopulation in half at random and constructed a training dataset (n = 2,347) and a validation dataset (n = 2,347). The details of the patient disposition are illustrated in Fig 1.

### Clustering-based diabetes subtyping method

We clustered our cohort using k-means clustering-based on selected baseline biomarkers and patient characteristics using the procedures described by Ahlqvist et al. [1]. These baseline characteristics are: HbA1c, age of diagnosis (AgeDiag), BMI, HOMA2-IR, and HOMA2-B. HOMA2 variables were calculated based on fasting plasma glucose (FPG) and f-Insulin. Ahlqvist et al. defined five diabetes subtypes, one of the subtypes corresponds to type 1 diabetes (severe autoimmune diabetes, SAID), and the other four subtypes corresponds to T2D. As type 1 diabetes was an exclusion criterion in the SAVOR-TIMI 53 trial, we expected to find four subtypes: severe insulin-deficient diabetes (SIDD), severe insulin-resistant diabetes (SIRD), mild obesity-related diabetes (MOD), mild age-related diabetes (MARD) clusters in our dataset. Thus, to identify the four subtypes defined by Ahlqvist et al., we have chosen k = 4 when conducting k-means clustering. This approach is consistent with others performed similar analyses with type 2 diabetes cohorts [3, 5, 8, 12].

The extreme values for HOMA2-IR were truncated (i.e., HOMA2-IR values greater than 20 were set to 20). All the variables were normalized (i.e., shifted and scaled) so that they have mean = 0 and standard deviations = 1. We applied the k-means clustering algorithm implemented in R version 3.5.1 [13] separately for male and female subpopulations of the training dataset. We then named each cluster center according to Ahlqvist et al. [1]. The best matching subtype name to each cluster center were determined as follows. First, the reference cluster centre matrix was created according to Ahlqvist et al. [1]. The reference cluster centre matrix was constructed by placing the vector of the normalized cluster centre (available in appendix of Ahlqvist et al. [1]) in each row of the matrix in the order of SIDD, SIRD, MOD and MARD. Then, a de novo cluster centre matrix was constructed, where the vectors of the normalized cluster centre from de novo clustering were used as the rows of this matrix. The row order of the de novo cluster centre matrix that minimizes the Frobenius norm of the differences between the reference cluster centre matrix was chosen. The matching subtype name was assigned to each row that corresponds to the vector of the de novo cluster centre.

The cluster centers (a reference for classification) identified in training dataset were applied to the validation dataset. In addition, to further assess the reproducibility of clustering when some of these variables are removed or replaced, we repeated the above analyses using the following sets of clustering variables: AgeDiag-HbA1c-HOMA2-IR, AgeDiag-HbA1c-f-Insulin, and AgeDiag-BMI-HbA1c.

#### Virtual patient datasets

To investigate the impact of correlations between the variables used for clustering on the reproducibility of clustering-based diabetes subtypes, we conducted a simulation study using virtual patient datasets (n = 100 000) containing simulated FPG, f-Insulin, HbA1c, AgeDiag and BMI. The simulated variables (FPG, f-Insulin, HbA1c, AgeDiag, BMI) were generated to follow a multivariate log-normal distribution. The log means and log-variances of the distribution were set to be the log-means and log-variances calculated from the training dataset from SAVOR-TIMI 53 trial. The means and standard deviations of these variables in the SAVOR-TIMI 53 trial can be found in the S1 Table. The qq-plots of log transformed variable confirming the log-normal distribution of the variables can be found in S1 Fig. We created virtual patient datasets with various correlations between clustering variables. The virtual patients with simulated FPG and f-Insulin values outside the defined range of HOMA2 (3.5<FPG<25mmol/L, 20<f-Insulin<400pmol/L) were removed from the virtual patient dataset. The HOMA2-IR and HOMA2-B were calculated using the simulated values for each virtual patient. Then the virtual patient dataset was divided into clustering-based diabetes subtypes following the procedure as described in the previous subsection. Finally, the reproducibility of these subtypes were assessed in order to evaluate the impact of the variable correlations.

### Assessment of reproducibility of clustering based diabetes subtypes

The reproducibility of the subtypes was investigated by conducting the k-means clustering as described above and by identifying the best matching subtype to each cluster. Then, the medians of clustering variables stratified by the subtypes were computed. The criterion for successful reproduction of each subtype was defined by median values satisfying the following criterion:

Severe insulin-deficient diabetes (SIDD): highest median HbA1c, lowest median HOMA2-B

Severe insulin-resistant diabetes (SIRD): Highest median HOMA2-IR and HOMA2-B

Mild obesity-related diabetes (MOD): highest median BMI

Mild age-related diabetes (MARD): highest median AgeDiag

In addition to applying these formal criteria, the box-plots of clustering variables similarly to Ahlqvist et al. [1] were made to illustrate the qualitative characteristics and the similarities to clusters presented by Ahlqvist et al. [1].

### Assessment of predictive utility of clustering-based diabetes subtypes

To explore if clustering-based diabetes subtypes can be used to predict outcome better than conventional risk scores, we compared the hazard ratios from the time to event analyses with respect to three endpoints: three-point MACE, eGFR reduction of more than 30% from the baseline, and introduction of new anti-diabetic medications.

We investigated the validation dataset (n = 2347) based on conventional risk classification related to each of the endpoints.

For the three-point MACE endpoint, we use the risk scoring proposed by Systematic COronary Risk Evaluation (SCORE) project [14] as a conventional risk classification. This scoring gives a numerical probability of 10-year risk estimates for fatal CV disease. The calculation of this score uses age, smoking status, systolic blood pressure and cholesterol measurements. Our dataset does not contain baseline lipid information, so cholesterol was set to 6mmol/L. SCORE has two different formulas depending on if the patients are in a high-risk country or a low-risk country. The high and low risk countries are mostly defined in the European Union member countries; on the other hand, the majority patients in SAVOR were from non-EU nations, thus for simplicity we used the formula used for high-risk countries. Based on the calculated probability of CV disease, we constructed risk subgroups with <5%, 5–10%, 10–15% and >15% probabilities.

For eGFR reduction of more than 30% from baseline, we used the risk classification proposed by in Kidney Disease: Improving Global Outcomes (KDIGO) clinical practice guideline [15]. This classification uses urine albumin-creatinine ratio (UACR) and eGFR. The level of risks are color coded by green, yellow, orange, and red and we used them as conventional risk groups for reduction of renal function.

The introduction of new anti-diabetic medication endpoint was compared with baseline HbA1c. Risk subgroups were created by dividing the patients in quantiles of baseline HbA1c.

Cox regression models were used on the validation dataset (n = 2347) to calculate the hazard ratios (HR) with 95% confidence interval of each endpoint for the clustering-based diabetes subtypes. Similarly, the HRs and confidence intervals were calculated for the subgroups constructed based on conventional risk classification for each endpoint. The predictive utility of diabetes subtypes and conventional classification was compared using the concordance statistics of the Cox regression model.

All analyses were done using R version 3.5.1 [13].

## Results

### Reproducibility of clustering-based type 2 diabetes subtypes in SAVOR-TIMI 53 trial-based dataset

**Fig 2:**
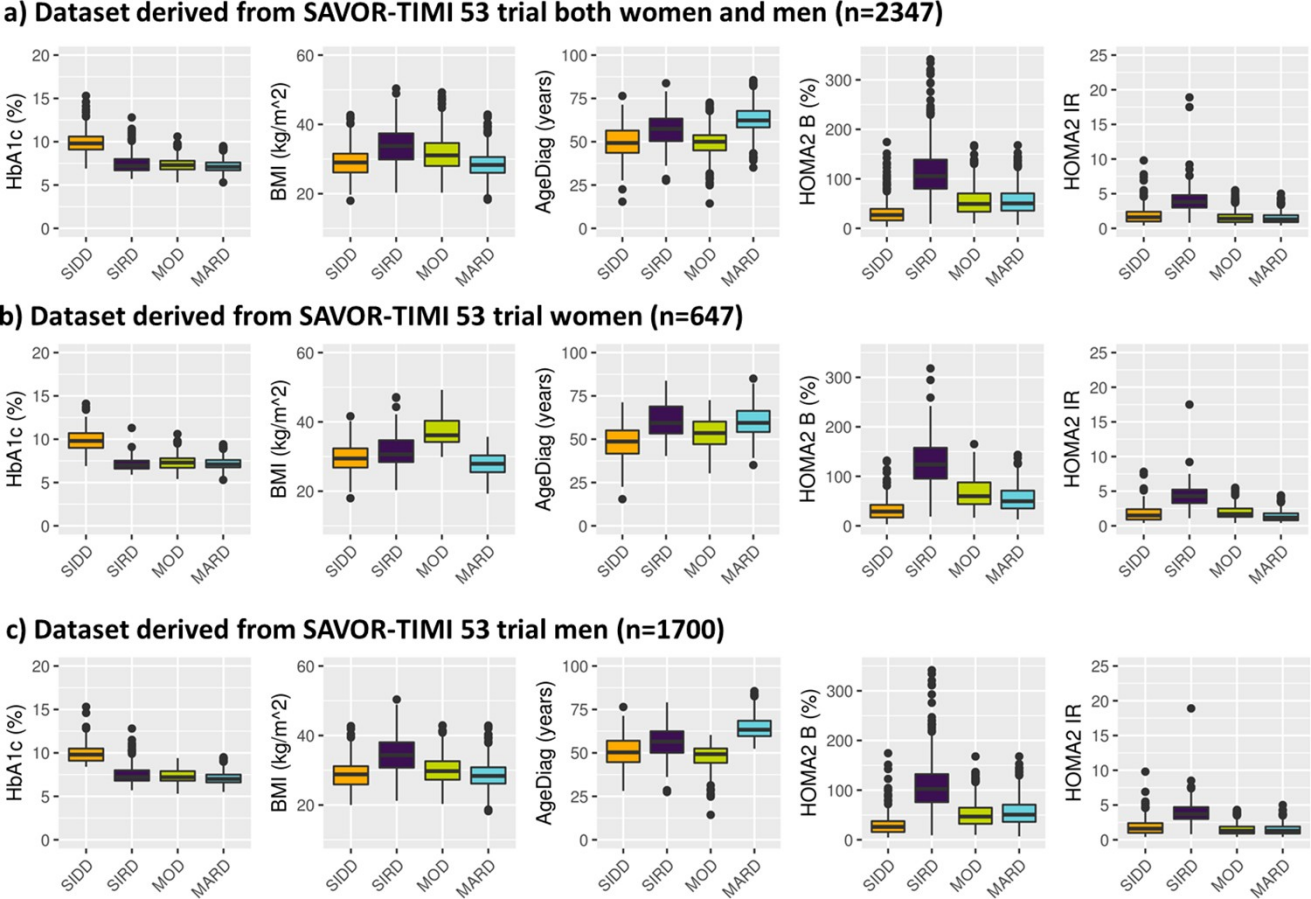
Distribution of baseline patient characteristics in SAVOR-TIMI 53 trial-based dataset used for the clustering and stratified by the resulting clusters. The clustering variable were prespecified including: HOMA2 B = homoeostatic model assessment 2 estimates of β-cell function, HOMA2 IR = homoeostatic model assessment 2 estimates of insulin resistance, HbA1c = Glycated hemoglobin, AgeDiag = age at diagnosis, and BMI = body mass index. SIDD = severe insulin-deficient diabetes, SIRD = severe insulin-resistant diabetes, MOD = mild obesity-related diabetes, MARD = mild age-related diabetes, HbA1c = Glycated hemoglobin, FPG = fasting plasma glucose, AgeDiag = age at diagnosis.

By applying the k-means clustering similarly to Ahlqvist et al., patients with T2D and established CV disease from SAVOR-TIMI 53 trial-based dataset were divided into four groups. As depicted in Fig 2A, the patient baseline characteristics of the clustering-based diabetes subtypes in SAVOR-TIMI 53 trial-based dataset were similar to what was reported for ANDIS and ADOPT [1, 5], except for BMI in MOD, which was lower in the current analysis. The differences in BMI distribution between the ANDIS and SAVOR-TIMI 53 cohorts could be explained by the gender imbalance of the SAVOR-TIMI 53 population (71.5% male in SAVOR-TIMI 53, while 55.6% male in ANDIS). In fact, the median BMI for SIRD patients were higher than MOD patients in men and the other way around in women (Fig 2B). This pattern was also observed in the ANDIS cohort. Hence, the over-representation of men in SAVOR-TIMI 53 population resulted in a higher BMI in patients in the SIRD group compared to patients in the MOD group.

**Fig 3:**
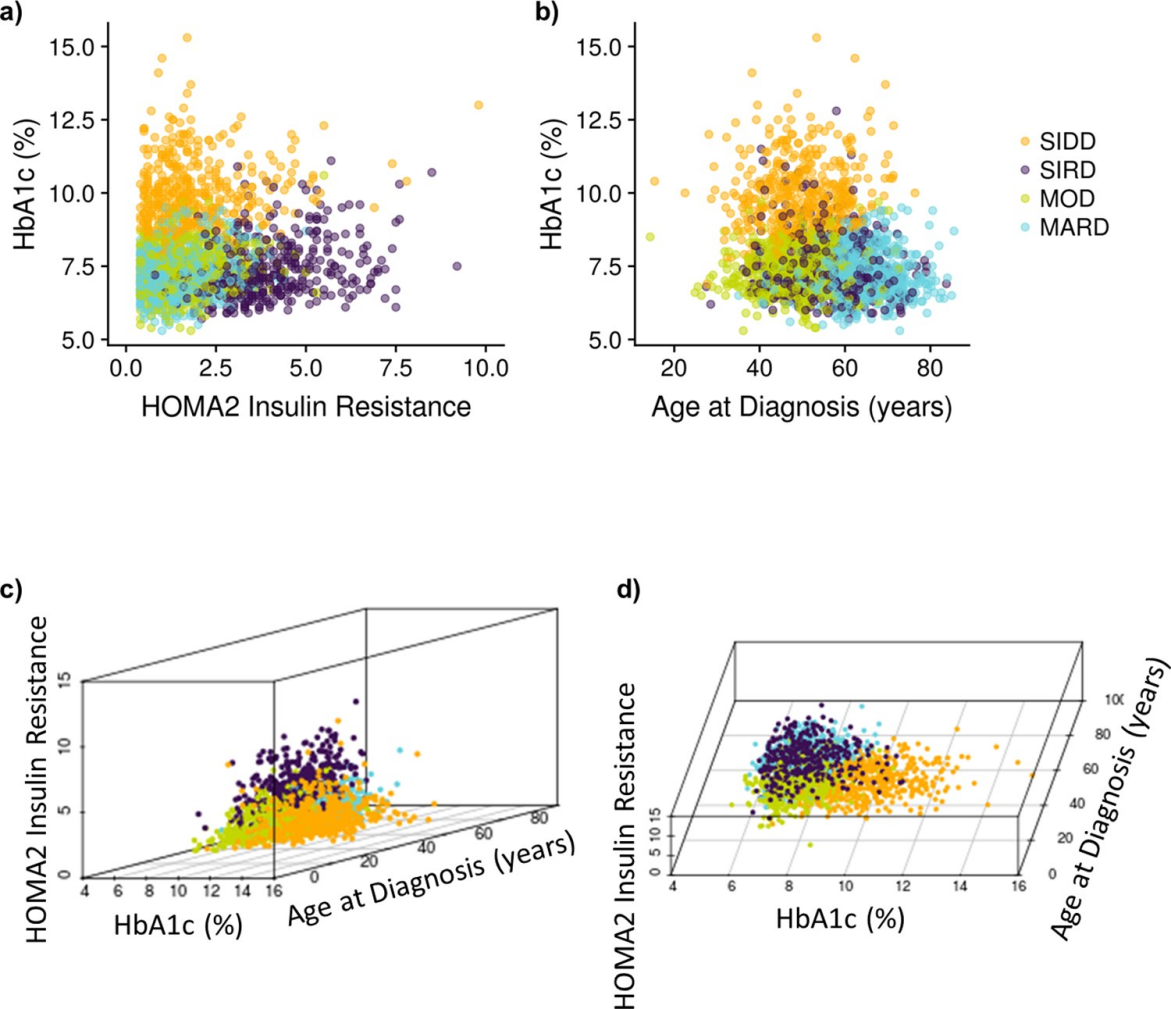
Two-dimensional and three-dimensional parameter distributions of the baseline patient characteristics of the dataset from SAVOR-TIMI 53 trial used for clustering. HbA1c, HOMA2 IR and age at diagnosis forms a tetrahedral shaped distribution. This gives a geometrical representation of the clustering. Given a dense tetrahedral shaped distribution, k-means clustering will form each cluster near each vertex of the tetrahedral. Patients can first be divided into SIRD patients and non-SIRD patients by HOMA2 IR and HOMA2 B. Then, non-SIRD patient can be divided by high and low HbA1c. If poor glycemic control, but less insulin resistant, the patient is insulin deficient; hence non-SIRD high-HbA1c patients can be classified as SIDD patients. Lastly, the non-SIRD-non-SIDD patients can be divided by age at diagnosis and those diagnosed at a later in life is clustered into MAD, indicating slow progression of the disease. MOD is the group that cannot be explained by insulin resistance, insulin deficiency, or ageing, but explained by obesity: HOMA2 Insulin Resistance = homoeostatic model assessment 2 estimates of insulin resistance, SIDD = severe insulin-deficient diabetes, SIRD = severe insulin-resistant diabetes, MOD = mild obesity-related diabetes, MARD = mild age-related diabetes.

**Fig 4:**
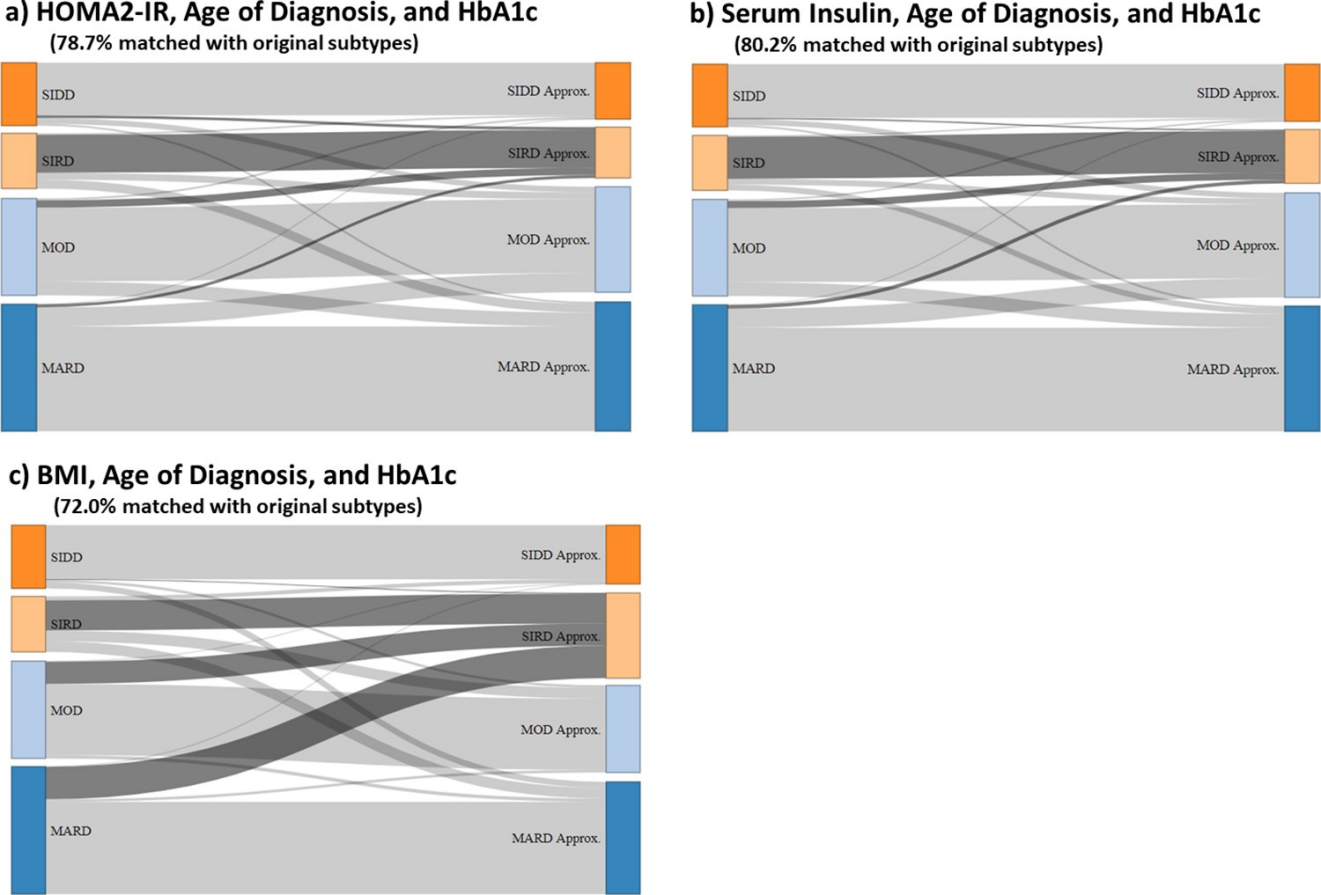
Sankey flow diagram showing the change in distribution comparing when the patients are subtyped according to Ahlqvist et al.’s original methodology (k-means clustering with HbA1c, HOMA2 IR, HOMA2 B, BMI, and age of diagnosis, shown to the left) with the distribution when the patients are subtyped by k-means clustering with reduced number of patient characteristics (shown to the right). HOMA2 IR = homoeostatic model assessment 2 estimates of insulin resistance, SIDD = severe insulin-deficient diabetes, SIRD = severe insulin-resistant diabetes, MOD = mild obesity-related diabetes, MARD = mild age-related diabetes. Approx. indicates the approximation of the original clustering-based diabetes subtypes using reduced number of patient characteristics.

To visualize the cluster-based diabetes subtypes, we made 3D plots of the variables used for clustering. Noteworthy, quantitative features of the variable distributions when plotted in 3D were that HbA1c, HOMA2-IR and AgeDiag formed a tetrahedral shaped distribution illustrating a geometrical representation of the clusters (Fig 3). Given the dense tetrahedral shaped distribution, k-means clustering formed each cluster near each vertex of the tetrahedral (Fig 3) indicating that the cluster-based subtyping may be approximated using these three variables. To further explore this observation, we conducted clustering only using HbA1c, HOMA2-IR and AgeDiag. As can be seen in Fig 4A, 78.7% of the patients got the same subtype as compared to the original clustering with all variables. Moreover, the approximated SIRD cluster included the majority of SIRD patients from the original subtype. In addition, f-Insulin could be used in place of HOMA2-IR. When using HbA1c, f-Insulin, and AgeDiag, 80.2% of the patients were subtyped to the same cluster as compared to when they were divided by k-means clustering with all variables (Fig 4B). We further investigated the 3 variables used by Kahkoska et al. [6] including HbA1c, BMI and AgeDiag. When using these variables 72% of the patients were subtyped to the same cluster as when they were subtyped by k-means clustering with all original variables (Fig 4C). However, the SIRD group became a mixture of the original MARD, MOD and SIRD patients when using HbA1c, BMI and AgeDiag (Fig 4C).

**Fig 5:**
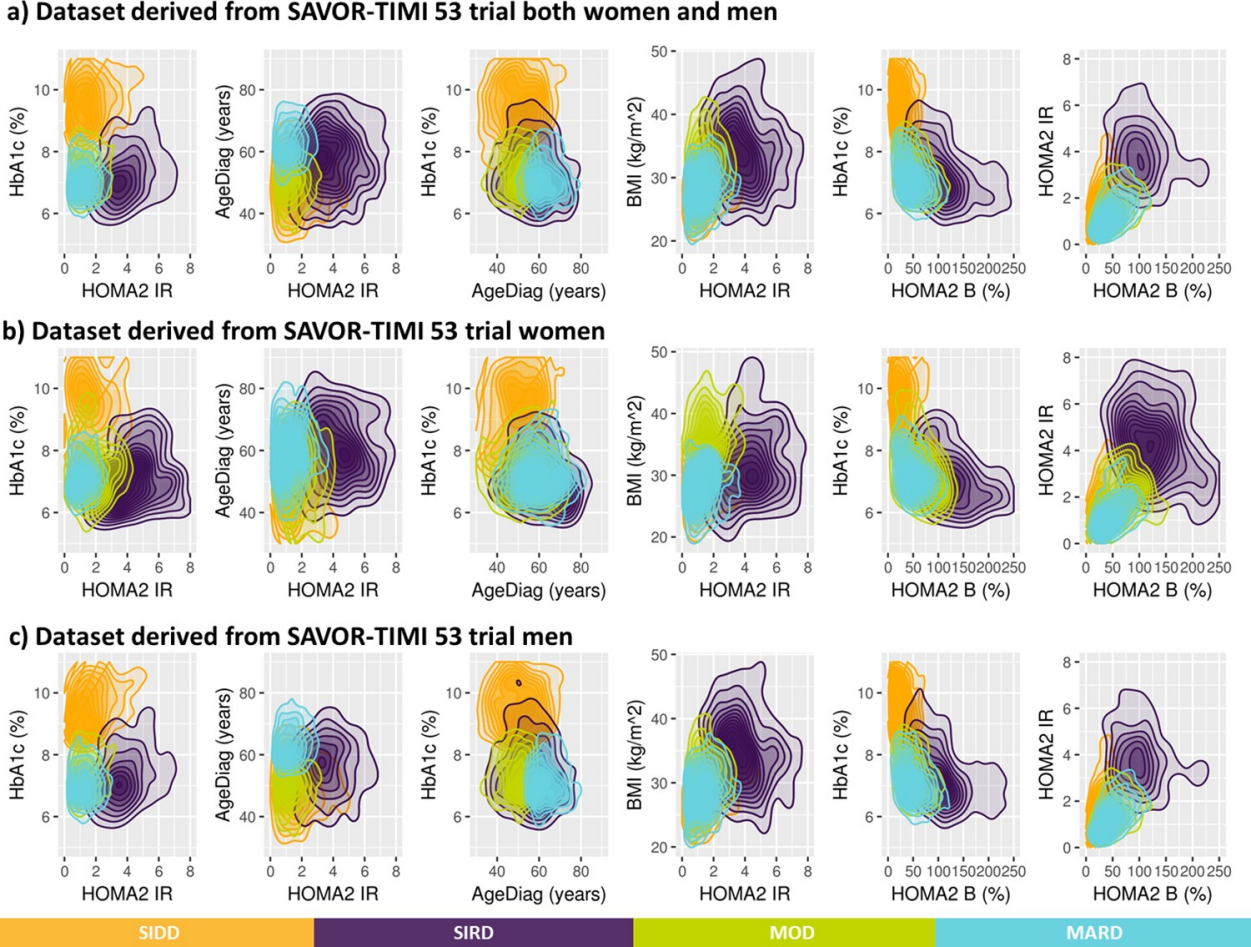
Two-dimensional density plots of distribution of patient characteristics following clustering. The clustering variables were prespecified including: HOMA2 B = homoeostatic model assessment 2 estimates of β-cell function. HOMA2 IR = homoeostatic model assessment 2 estimates of insulin resistance, HbA1c = Glycated hemoglobin, AgeDiag = age at diagnosis, and BMI = body mass index. The number of clusters is prespecified to be four and named after diabetes subtypes defined by Ahlqvist et al.: SIDD = severe insulin-deficient diabetes, SIRD = severe insulin-resistant diabetes, MOD = mild obesity-related diabetes, MARD = mild age-related diabetes.

Motivated by this tetrahedral shape, 2D density plots were created (Fig 5). The 2D distribution of HbA1c and HOMA2-IR shows that SIDD and SIRD are separated while MOD and MARD are overlapping. In the 2D density plot of AgeDiag and HOMA2-IR (Fig 5B and 5C), a clear separation between MOD and MARD was observed for men, but not for women. Another sex difference were the different shapes of the BMI-HOMA2-IR distributions between men and women with respect to MOD and MARD. The difference in correlation coefficients between BMI and HOMA2-IR between men and women, (0.38 and 0.24, respectively) may contribute to the difference in separation between MOD and MARD in men and women. These observations suggest that the correlations between variables and the shapes of the distributions created by the correlations may impact the reproducibility of the clustering-based diabetes subtypes.

**Fig 6:**
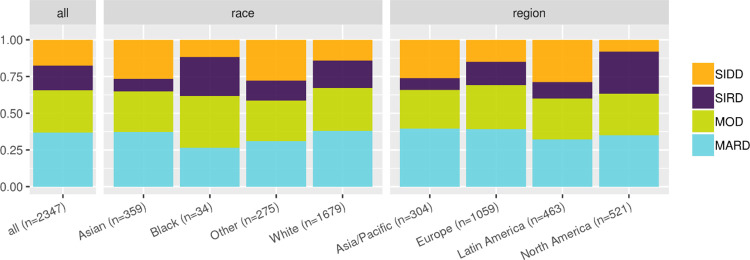
Distribution of the clustering-based diabetes subtypes stratified by the region and race. SIDD = severe insulin-deficient diabetes, SIRD = severe insulin-resistant diabetes, MOD = mild obesity-related diabetes, MARD = mild age-related diabetes.

The patient distribution of the clustering-based diabetes subtypes in the SAVOR-TIMI 53 trial-based validation-dataset were as follows: SIDD 18%, SIRD 17%, MOD 29%, and MARD 37%. As seen in Fig 6, the proportion of MOD and MARD are consistent across races and regions. However, there were more SIRD patients than SIDD patients in North America, while in Europe, there was approximately the same number of SIRD and SIDD patients. In Asia/Pacific and Latin America, there were less SIRD patients than SIDD patients. This pattern has been reported previously in Chinese and Japanese cohorts [3, 7].

### Assessment of the impact of correlations for the reproducibility of clustering-based diabetes subtypes (investigation using virtual patient datasets)

It is well established that some of the variables used in clustering-based diabetes subtypes are correlated and we hypothesized that the correlations may play an important role in the reproducibility of the diabetes subtypes across different patient cohorts. To test this hypothesis, we created virtual patient datasets with various variable correlations.

The three strongest correlations between patient characteristic variables and biomarkers in the SAVOR-TIMI 53 trial-based data set (FPG, f-Insulin, AgeDiag, HbA1c, BMI) were: HbA1c-FPG (correlation coefficient all: 0.62, women: 0.63, men: 0.61), BMI-f-Insulin (all: 0.34, women: 0.24, men: 0.39) and AgeDiag-HbA1c (all: -0.24, women: -0.26, men: -0.23). It is noteworthy that the correlations between BMI and f-Insulin were stronger in men than women and is related to the gender difference in fat distribution [16].

To investigate and visualize the impact of the correlations four virtual patient datasets were created with a stepwise introduction of correlations as follows: 1) no correlations between variables (FPG, f-Insulin, AgeDiag, HbA1c, BMI), 2) a strong correlation between HbA1c and FPG (correlation coefficient of 0.5), 3) a strong correlation between HbA1c and FPG (correlation coefficient of 0.5) and mild correlations between BMI-f-Insulin (correlation coefficient of 0.25) and AgeDiag-HbA1c (correlation coefficient of -0.25), 4) strong correlations between HbA1c-FPG and BMI-f-Insulin (correlation coefficient 0.5) and a mild correlation between AgeDiag-HbA1c (correlation coefficient of -0.25).

**Fig 7:**
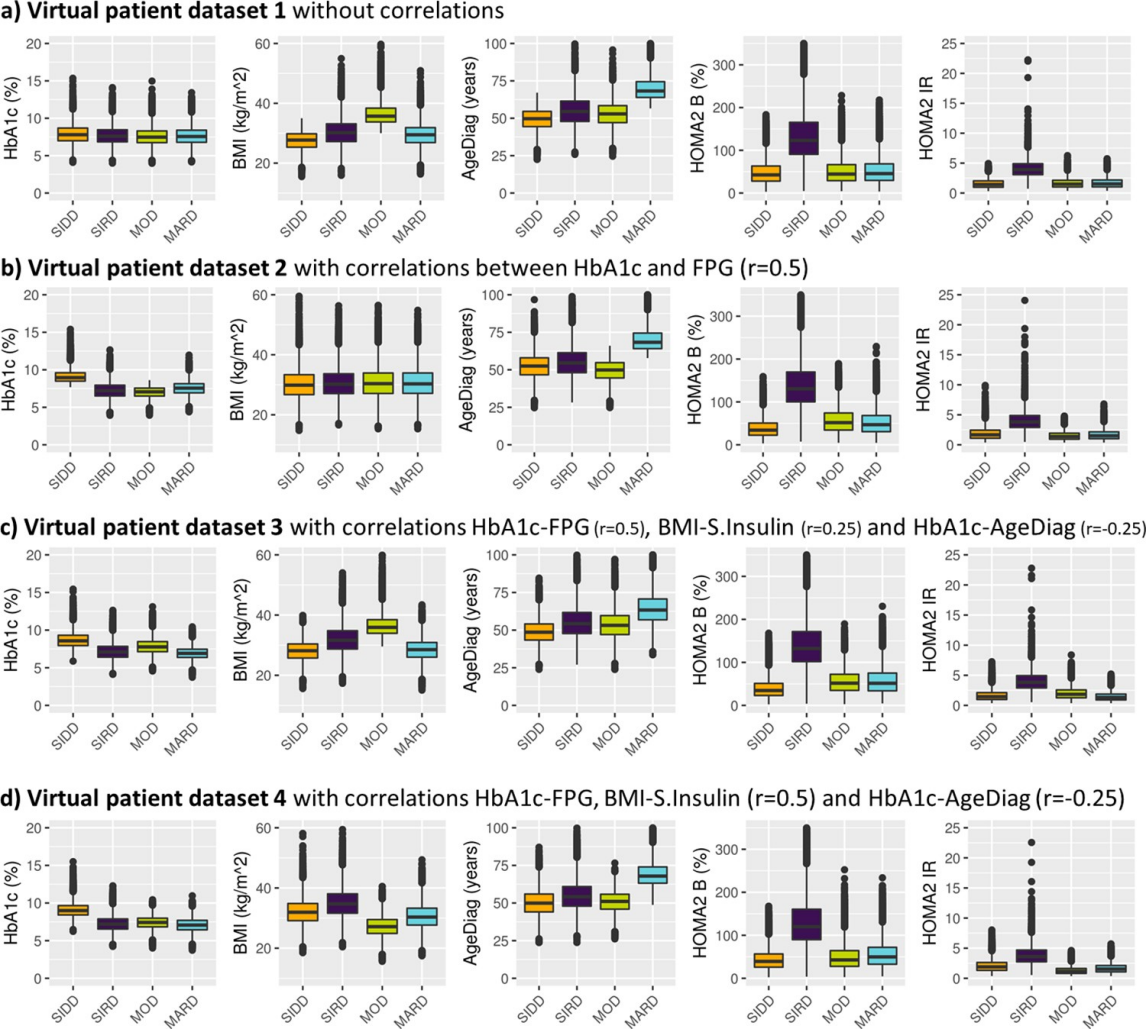
Clustering based on a virtual patient dataset not including (panel a) or including correlations between variables (panel b, c and d). Distribution of baseline patient characteristics used for the clustering are stratified by the resulting clusters. The clustering variables were prespecified including: HOMA2 B = homoeostatic model assessment 2 estimates of β-cell function. HOMA2 IR = homoeostatic model assessment 2 estimates of insulin resistance, HbA1c = Glycated hemoglobin, AgeDiag = age at diagnosis, and BMI = body mass index. The number of clusters is prespecified to be four and named after diabetes subtypes defined by Ahlqvist et al.: SIDD = severe insulin-deficient diabetes, SIRD = severe insulin-resistant diabetes, MOD = mild obesity-related diabetes, MARD = mild age-related diabetes.

**Fig 8:**
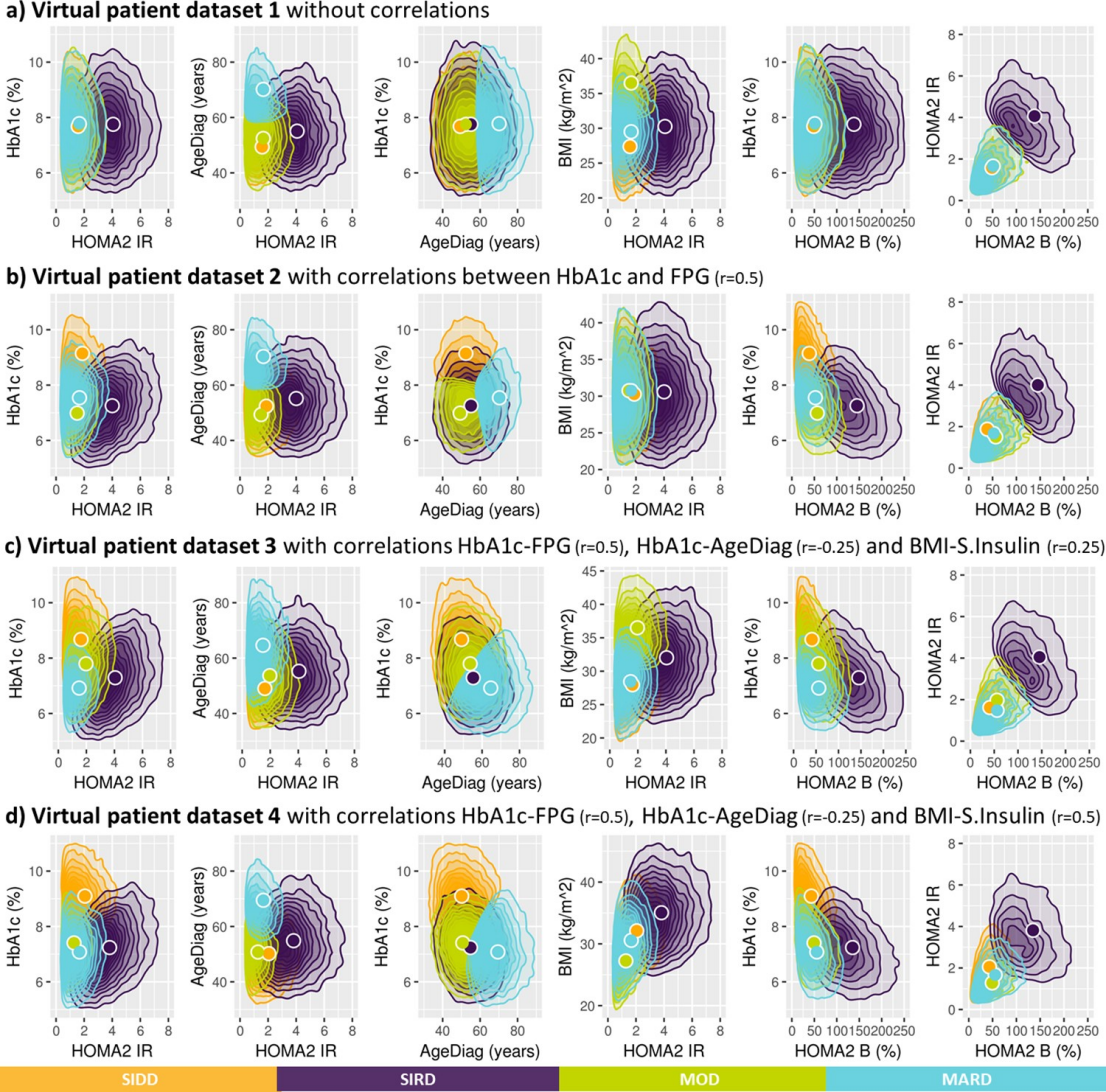
Two-dimensional density plots of distribution of patient characteristics following clustering. The clustering variables were prespecified including: HOMA2 B = homoeostatic model assessment 2 estimates of β-cell function. HOMA2 IR = homoeostatic model assessment 2 estimates of insulin resistance, HbA1c = Glycated hemoglobin, AgeDiag = age at diagnosis, and BMI = body mass index. The number of clusters is prespecified to be four and named after diabetes subtypes defined by Ahlqvist et al.: SIDD = severe insulin-deficient diabetes, SIRD = severe insulin-resistant diabetes, MOD = mild obesity-related diabetes, MARD = mild age-related diabetes. Circles indicate the means (centroids) that were found by the k-mean clustering algorithm.

When k-means clustering was applied to the virtual patient dataset without any correlations between HbA1c, BMI, AgeDiag, f-Insulin and FPG (i.e., Virtual patient dataset 1). This clustering resulted in subgroups with SIRD, MOD, and MARD characteristics, but not SIDD (see Fig 7A). One noteworthy characteristic in the variable distributions was the correlation between HOMA2-IR and HOMA2-B (correlation coefficient: 0.496, Fig 8A). Although the original variables (f-Insulin and FPG) were not correlated, as the result of HOMA2 calculation, HOMA2-IR and HOMA2-B formed correlated distribution. When the virtual patient population is generated with a correlation of 0.5 between HbA1c and FPG (i.e., Virtual patient dataset 2), the subpopulations with the characteristics as SIRD, MARD, and SIDD were generated, but not MOD (Fig 7B). In Fig 8B we can observe the similarly shaped distributions between HbA1c and HOMA2 variables that were observed in SAVOR-TIMI 53 trial-based dataset was reproduced when the correlations between HbA1c and FPG was added. As a result of this shape of the distribution between HOMA2 variables and HbA1c, we were able to reproduce SIDD.

Next, the virtual patient dataset with correlations of 0.25 between BMI-f-Insulin and -0.25 between HbA1c-AgeDiag were used (i.e., Virtual patient dataset 3). Using this virtual patient dataset with these correlation all four subgroups could be reproduced (Fig 7C). As can be seen in S2 Fig, the distribution of variables and cluster divisions of Virtual patient dataset 3 are very similar to the women in SAVOR-TIMI 53 trial-based dataset. In addition, when adding correlations that is more similar to men in SAVOR-TIMI 53 trial, 0.5 and -0.25 between BMI-f-Insulin and HbA1c-AgeDiag, respectively (Virtual patient dataset 4), SIDD, SIRD and MARD were reproduced, but not MOD (Fig 7D). This finding is consistent with what was observed in men in the SAVOR-TIMI 53 trial-based dataset (Fig 2C). In addition, as can be seen in S3 Fig, overall distributions of the variables and cluster divisions of this virtual patient dataset are qualitatively similar to the men in SAVOR-TIMI 53 trial-based dataset. Thus, the 2D plots illustrating BMI and HOMA2-IR in men and women in SAVOR-TIMI 53 trial-based dataset (Fig 5B and 5C) were reproduced by changing the degree of correlation between BMI and f-Insulin (Fig 7C and 7D). Consequently, the lack of reproducibility of MOD in men (hence in overall population) in SAVOR-TIMI 53 trial-based dataset can be explained from different BMI and f-insulin correlations in men and women.

### Predictive utility of clustering-based diabetes subtypes for diabetic disease progression and diabetes related complications

**Fig 9:**
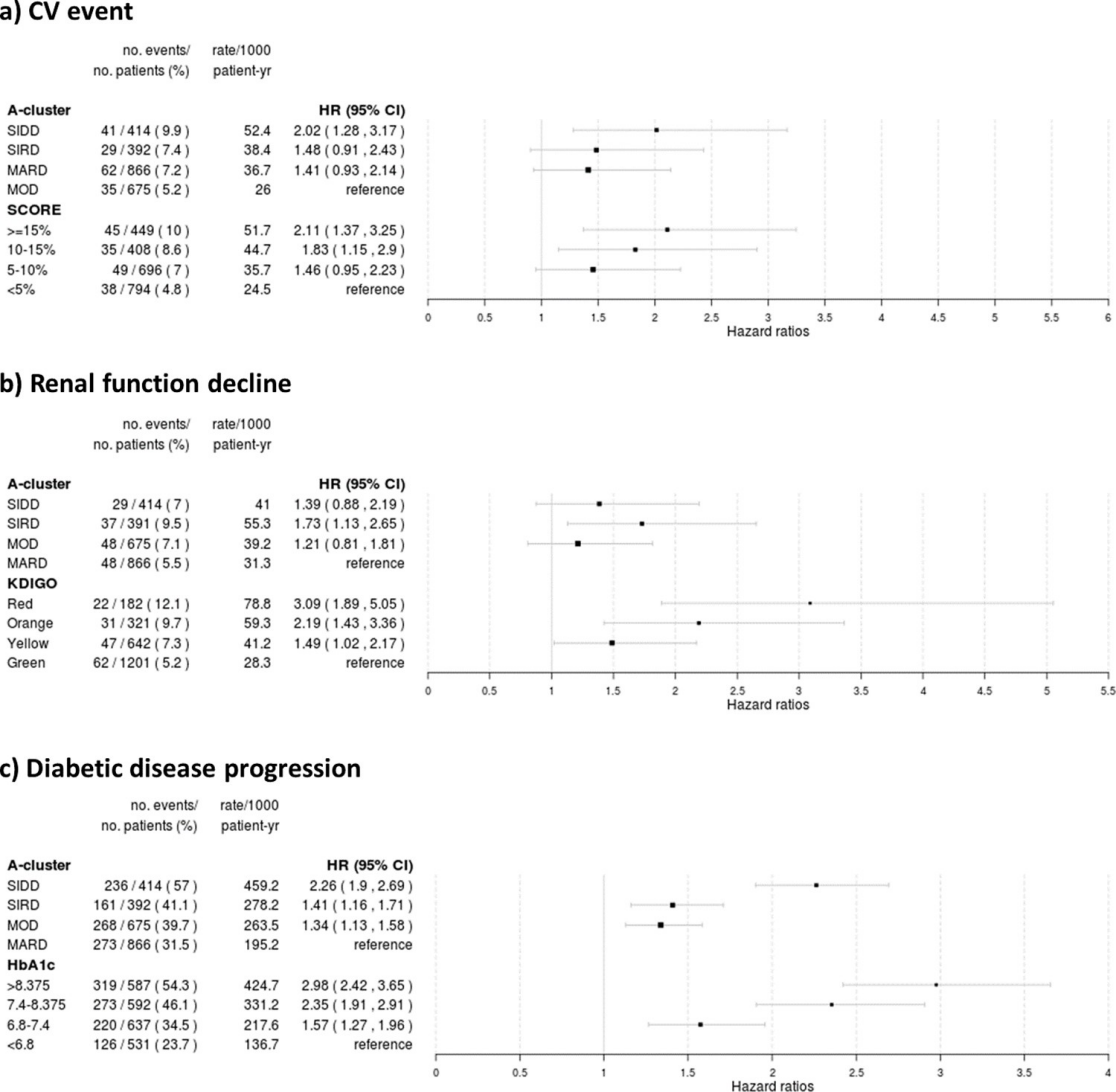
Risk estimations for CV events (panel a), renal function decline (panel b) and diabetes disease progression based (panel c). The results are presented as Hazard ratios (HR) with 95% confidence interval (CI) of various endpoints in the validation dataset (n = 2347) estimated by Cox proportional hazard model, according to clusters and clustering methods: SIDD = severe insulin-deficient diabetes, SIRD = severe insulin-resistant diabetes, MOD = mild obesity-related diabetes, MARD = mild age-related diabetes, A-cluster = cluster-based subtyping following method proposed by Ahlqvist et al., SCORE = risk prediction based on Systematic COronary Risk Evaluation project, KDIGO = risk prediction based on Kidney Disease: Improving Global Outcomes project.

The hazard ratios and 95% confidence intervals for three-point MACE of clustering-based diabetes subtypes: SIDD, SIRD, MARD as compared to MOD were 2.02 (1.28–3.17), 1.48 (0.91–2.43), and 1.41 (0.93–2.14), respectively. The conventional CV risk classification (SCORE): > = 15%, 10–15%, 5–10% 10-year risk estimates for fatal CV disease compared to the lowest risk group were 2.11 (1.37–3.25), 1.83 (1.15–2.90) and 1.46 (0.95–2.23), respectively (Fig 9A). The concordance statistics for clustering-based diabetes subtypes and SCORE were 0.566 and 0.573, respectively. This finding indicate that the conventional risk classification defined by SCORE performed slightly better than clustering-based diabetes subtypes when predicting three-point MACE in patients with T2D and established CV disease.

The hazard ratios and 95% confidence intervals for reduction in kidney function of clustering-based diabetes subtypes: SIDD, SIRD, MOD as compared to MARD were 1.39 (0.88–2.19), 1.73 (1.10–2.65), and 1.21 (0.81–1.81), respectively. The conventional renal risk classification (KDIGO): Red, Orange, Yellow group compared to the lowest risk group (Green group) were 3.09 (1.89–5.05), 2.19 (1.43–3.36) and 1.49 (1.02–2.17), respectively (Fig 9B). The concordance statistics for clustering-based diabetes subtypes and KDIGO were 0.540 and 0.616, respectively, indicating that the conventional risk classification defined by KDIGO better predict reduction in kidney function than clustering-based diabetes subtypes in patients with T2D and established CV disease.

The hazard ratios and 95% confidence intervals for diabetic disease progression of clustering-based diabetes subtypes: SIDD, SIRD, MOD as compared to MARD were 2.26 (1.90–2.69), 1.41 (1.16–1.71), and 1.34 (1.13–1.58), respectively. When using quartiles of HbA1c to predict disease progression compared to the lowest risk group (lowest quartile of HbA1c) the HR and 95% confidence intervals were 2.98 (2.42–3.65), 2.35 (1.91–2.91) and 1.57 (1.27–1.96), respectively (Fig 9C). The concordance statistics for clustering-based diabetes subtypes and quartiles of HbA1c were 0.575 and 0.607, respectively, indicats that the quartiles of HbA1c better predicts diabetes disease progression than clustering-based diabetes subtypes in patients with T2D and established CV disease.

## Discussion

We have shown that the proposed classification of diabetic patients into clustering-based diabetes subtypes as defined by Ahlqvist et al. [1, 17] using k-means clustering algorithm of newly diagnosed diabetes patients can to a large extent be replicated in a subset of patients with T2D and established CV disease from the large CV outcome study, SAVOR-TIMI 53. It is worthy to note that this shows the reproducibility of subtype classification specified by Ahlqvist et al. even if we apply the same methodology to a segment of T2D patients with established CV disease. Also, we have shown that instead of including the five patient characteristics and biomarkers chosen by Ahlquist et al. (AgeDiag, BMI, HbA1c, HOMA2-IR and HOMA2-B), using only three variables AgeDiag, HbA1c, and HOMA2-IR (or f-Insulin) result in a similar subtype classification. The general trend of the distribution of subtypes, and patient characteristics in each subtype were similar to what have been reported previously in wide range of patient cohorts [1, 3–8, 18]. Interestingly, similar clustering was possible using virtual patient datasets based on the multivariate log-normal distribution and clinically relevant known correlations between the variables. We found that SIDD, SIRD, and MARD can be reproduced from the log-normal distribution of BMI, HbA1c, AgeDiag, f-Insulin and FPG with a positive correlation between HbA1c and FPG. MOD can also be reproduced by adding two correlations of low degree between BMI-f-Insulin and HbA1c-AgeDiag. The lack of reproducibility of MOD in men in the SAVOR-TIMI 53 trial-based dataset was explained by the strength of the correlation between BMI and f-Insulin. Based on these analyses using virtual patient datasets generated from the normal distribution, we postulate that the consistent replication of these subtypes across wide-range of patient cohorts can mostly be explained by the consequence of applying k-means clustering to the dataset with well-established correlations and non-linear dependencies between variables that are structurally generated through transformation of FPG and f-Insulin to HOMA2 values. In addition, the elbow method based on the sum of squares distance and the Silhouette width analyses did not suggest four to be the optimal number of clusters (see S4 Fig), indicating that reproducibility of the subtypes may not be due to a consistent finding of optimal clusters.

In our analysis based on the segment of T2D patients with established CV disease, it was observed that SIDD had the highest risk for MACE. Ahlqvist et al. reported no statistically significant difference in terms of time to coronary events, while unadjusted findings indicated the SIRD had the highest risk [1]. In DEVOTE and LEADER trials, the SIDD-like subtype had the highest risk for MACE events [6]. In SUSTAIN-6 and RECORD cohort, there was almost no difference in the risk for CV events between the clustering-based diabetes subtypes [5, 6]. It is possible that MACE was less well associated with subtypes in DEVOTE, LEADER and SUSTAIN-6 trials since HOMA2-IR variables were not included, and therefore, there was a less clear definition of SIRD (Fig 4C). However, our analysis show that even with the addition of HOMA2 values, SIDD has the highest risk for MACE. Also DEVOTE, LEADER and SUSTAIN-6 included patients with established CV disease [6], being more similar to the SAVOR-TIMI 53 cohort; thus, it is possible to hypothesize that the SIDD has the highest risk among patients with T2D and established CV disease. Nevertheless, clustering-based diabetes subtypes did not show any advantage over the conventional risk classification (SCORE) in this group of patients with T2D even though we were lacking some variables to accurately calculate SCORE and, moreover, SCORE was constructed for long-term prediction of risk. It must also be pointed out that our analyses are based on a selected population of T2D patients with established CV disease; thus, these results may not extend to newly diagnosed T2D patients or a general T2D patient population. That is to say clustering-based diabetes subtypes defined by Ahlqvist et al. may result in a good risk prediction model for newly diagnosed diabetic patients but does not predict CV risk better than an conventional CV risk score in patients with a history of diabetes and established CV disease.

For the renal function reduction, our analysis of diabetes patients with established CV disease showed that the risk for a decrease in eGFR decrease was highest in the SIRD group as compared to the other diabetes subtypes. This is consistent with what reported by Ahlqvist et al. [1]. However, the risk for reduction in renal function can also be predicted by the risk scoring proposed in KDIGO clinical guideline using UACR and eGFR. The better prediction with KDIGO as compared to clustering-based diabetes subtypes is likely explained the fact the clustering-based subtyping does not make any use of renal function related variables.

We were able to reproduce the pattern of diabetic disease progression similarly to what was presented by Ahlqvist et al.; SIDD having the fastest progression and MARD the slowest progression, while SIRD and MOD progress similarly. However, the diabetic disease progression can also be predicted by using baseline HbA1c levels, which is not surprising since addition of new anti-diabetic drugs are largely based on this variable. Our exploratory analyses have several limitations. Although we have carefully designed the analysis by dividing our cohort into training and validation, our analysis is based on the dataset from one large cardiovascular outcome trial; and does not represent general T2D diabetes populations. Therefore, it will be desirable to validate our results further using other diabetes cohorts. Furthermore, we excluded patients on insulin therapy because of the potential impact on HOMA2 calculations.

## Conclusion

We conclude that clustering-based diabetes subtypes defined by Ahlqvist et al. can be reproduced in the segment of T2D patients with a history of CV disease. The robust and consistent reproduction of diabetes subtypes was explained by the clinically known correlations between related variables used in clustering. These subtypes were not better than conventional risk scores to predict risk of recurrent cardiovascular events or progression of renal disease in patients with T2D and established CV disease.

## Supporting information

S1 Fig qqplots of the variables used to create virtual patient dataset. (TIF)

S2 Fig Comparison of variable distributions between the women in SAVOR-TIMI 53 trial based dataset v.s. the virtual patient dataset. For the ease of comparison, Figs 2B, 5B, 7C, and 8C are placed side by side.
(TIF)

S3 Fig Comparison of variable distributions between the women in SAVOR-TIMI 53 trial based dataset v.s. the virtual patient dataset. For the ease of comparison, Figs 2C, 5C, 7D, and 8D are placed side by side.
(TIF)

S4 Fig Plots of within sum of suqares and Silhouette width both for SAVOR-TIMI 53 trial dataset and virtual patient dataset. The elbow method based on the total within the sum of square indicated the optimal number of clusters to be one for both SAVOR-TIMI 53 trial-based and virtual patient datasets. The average Silhouette width-based analyses indicated two to be the optimal number of clusters for the SAVOR-TIMI 53 trial-based and virtual patient datasets. Note that Silhouette width is not defined for the case of one cluster; hence it has indicated the minimum possible number of clusters to be the optimal number of clusters. These analyses suggest that the clusters, at least in the traditional sense, do not exist in the SAVOR-TIMI 53 trial dataset nor the virtual patient dataset.
(TIF)

S1 Table Summary statistics used to generate virtual patient dataset. (PDF)
