# Supplementary figures and images for: Assessing reproducibility and utility of clustering of patients with type 2 diabetes and established CV disease (SAVOR -TIMI 53 trial)
Source: PLoS One. 2021 Nov 19;16(11):e0259372. doi: 10.1371/journal.pone.0259372 (PMC8604302; doi:10.1371/journal.pone.0259372)

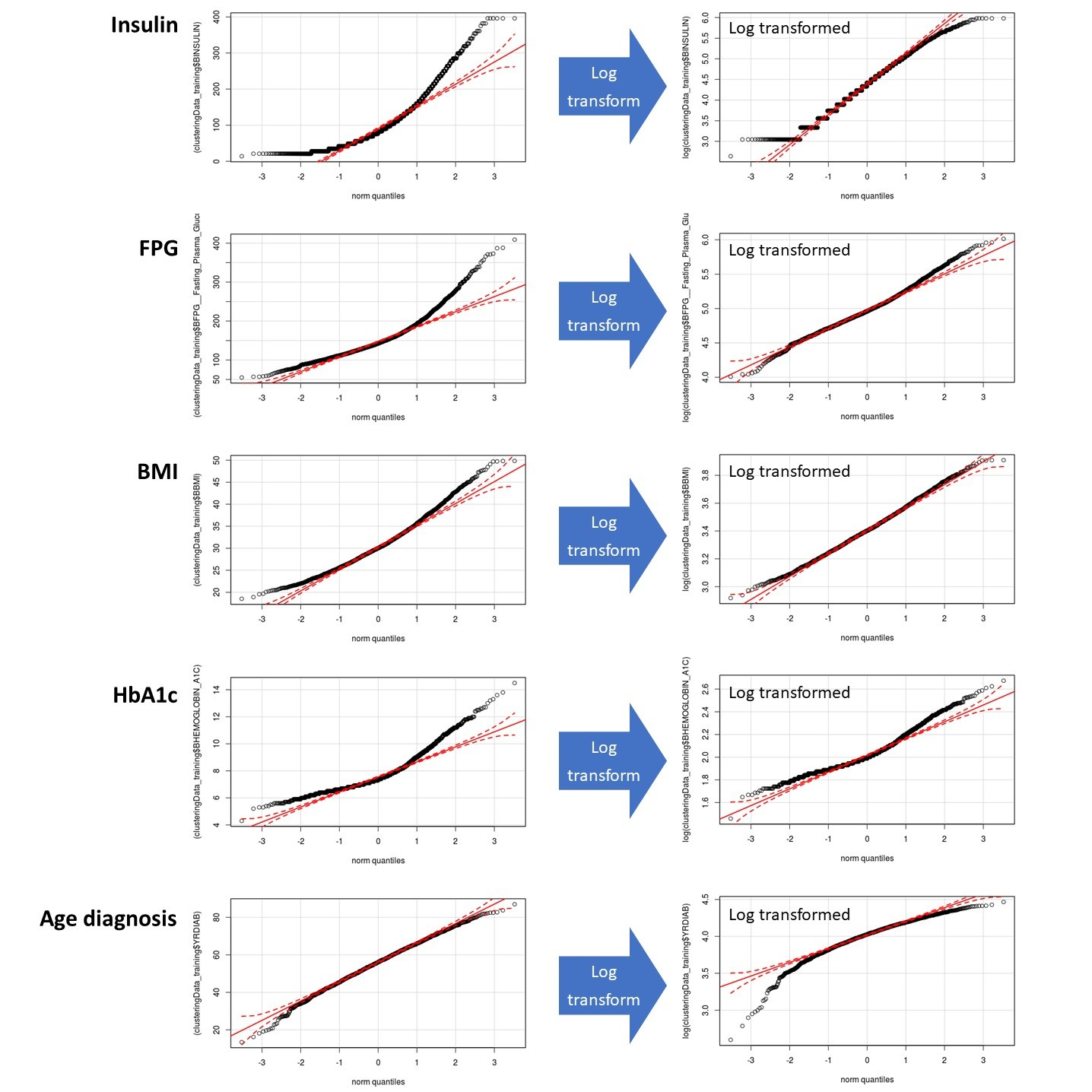

Supplement: S1 Fig — (TIF) [file pone.0259372.s001.tif]

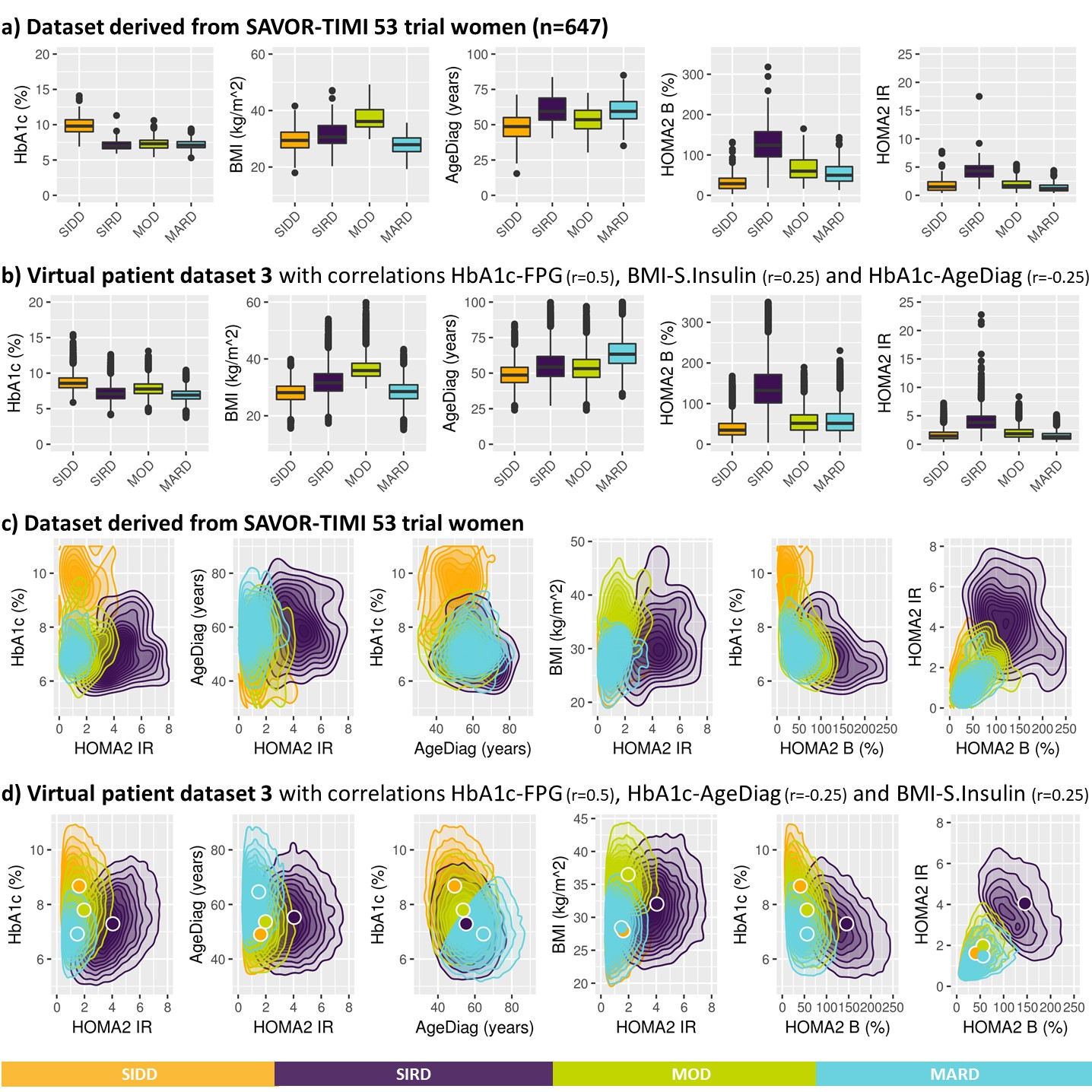

Supplement: S2 Fig — For the ease of comparison, Figs 2B, 5B, 7C, and 8C are placed side by side. (TIF) [file pone.0259372.s002.tif]

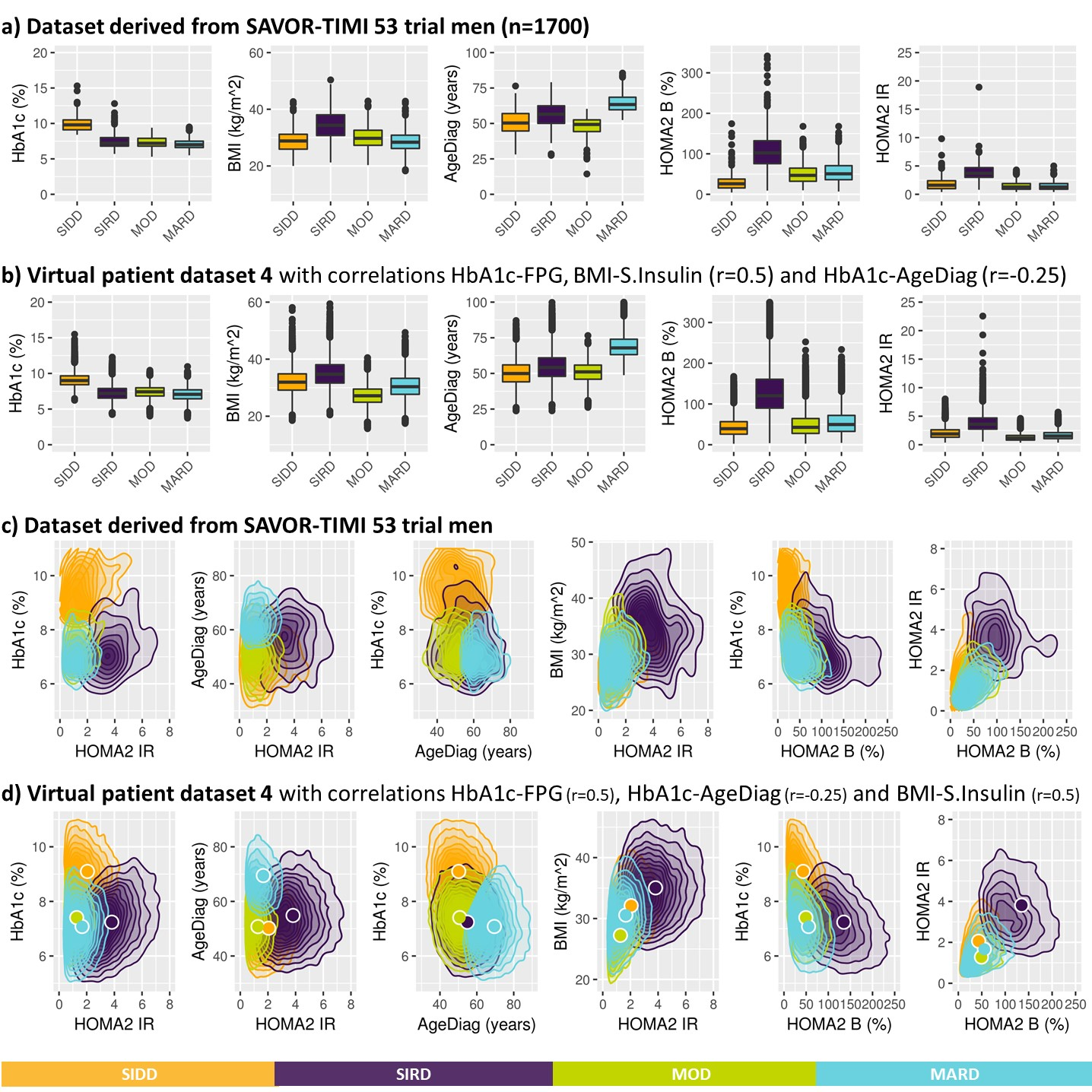

Supplement: S3 Fig — For the ease of comparison, Figs 2C, 5C, 7D, and 8D are placed side by side. (TIF) [file pone.0259372.s003.tif]

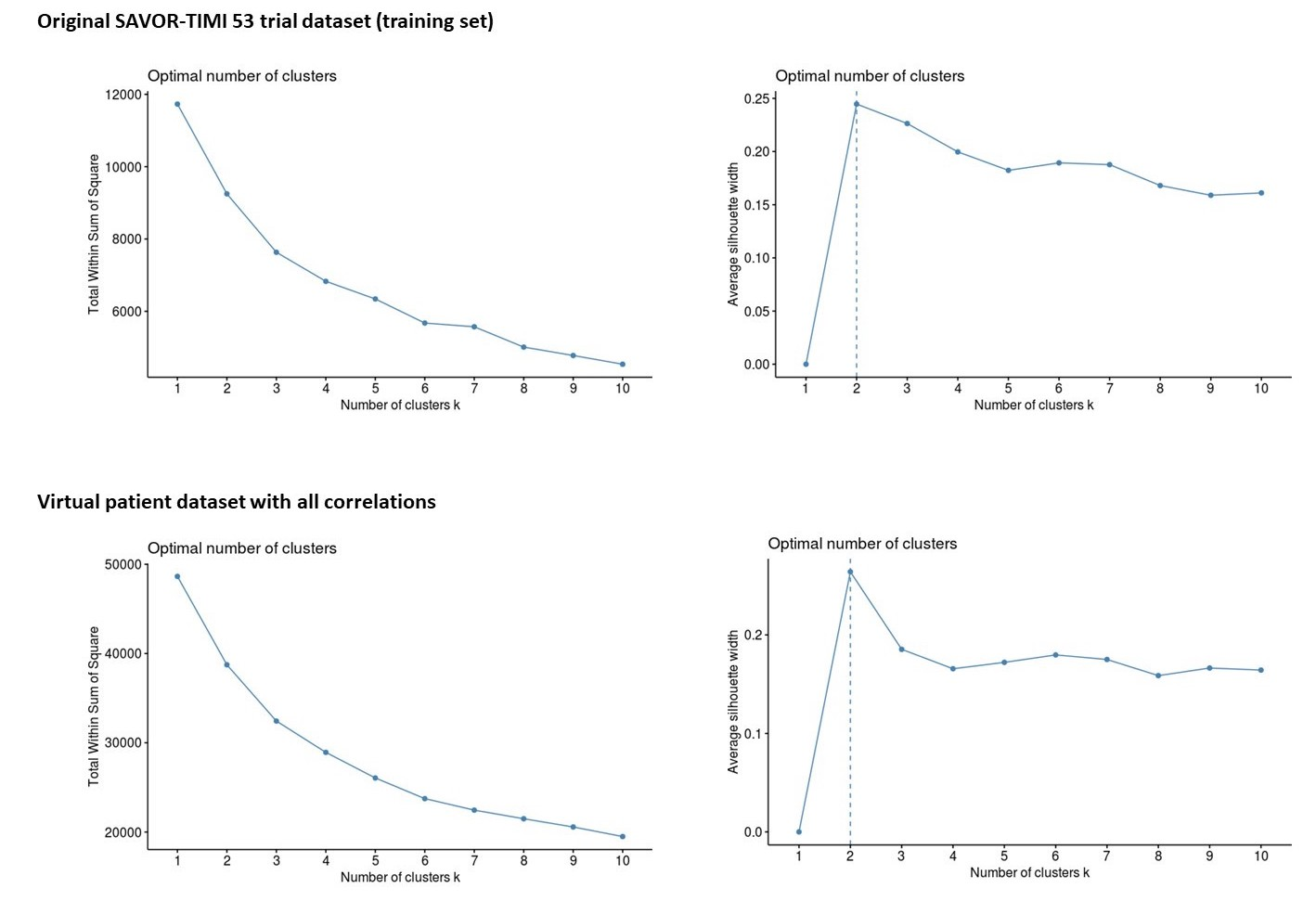

Supplement: S4 Fig — The elbow method based on the total within the sum of square indicated the optimal number of clusters to be one for both SAVOR-TIMI 53 trial-based and virtual patient datasets. The average Silhouette width-based analyses indicated two to be the optimal number of clusters for the SAVOR-TIMI 53 trial-based and virtual patient datasets. Note that Silhouette width is not defined for the case of one cluster; hence it has indicated the minimum possible number of clusters to be the optimal number of clusters. These analyses suggest that the clusters, at least in the traditional sense, do not exist in the SAVOR-TIMI 53 trial dataset nor the virtual patient dataset. (TIF) [file pone.0259372.s004.tif]
