# Supplementary material for: Assessing reproducibility and utility of clustering of patients with type 2 diabetes and established CV disease (SAVOR -TIMI 53 trial)
Source: PLoS One. 2021 Nov 19;16(11):e0259372. doi: 10.1371/journal.pone.0259372 (PMC8604302; doi:10.1371/journal.pone.0259372)
Supplement: S1 Table — (PDF) [file pone.0259372.s005.pdf]

**SAVOR TIMI-53 training dataset** (N = 2347)	
**Age of T2D diagnosis (years)**	
min	40
max	95
mean (sd)	64.36 ± 9.22
mean (sd) of log transformed	4.15 ± 0.15
**HbA1c (%)**	
min	4.3
max	14.5
mean (sd)	7.73 ± 1.32
mean (sd) of log transformed	2.03 ± 0.16
**BMI (kg/m^2)**	
min	18.51071
max	49.84044
mean (sd)	30.60 ± 5.13
mean (sd) of log transformed	3.41 ± 0.16
**FPG (mg/dL)**	
min	55
max	409
mean (sd)	152.22 ± 47.31
mean (sd) of log transformed	4.98 ± 0.29
**f-Insulin (pmol/L)**	
min	13.99
max	396
mean (sd)	98.90 ± 68.04
mean (sd) of log transformed	4.38 ± 0.67
